# Overexpression of Full-Length Centrobin Rescues Limb Malformation but Not Male Fertility of the Hypodactylous (*hd*) Rats

**DOI:** 10.1371/journal.pone.0060859

**Published:** 2013-04-08

**Authors:** František Liška, Claudia Gosele, Elena Popova, Blanka Chylíková, Drahomíra Křenová, Vladimír Křen, Michael Bader, Laura L. Tres, Norbert Hubner, Abraham L. Kierszenbaum

**Affiliations:** 1 Institute of Biology and Medical Genetics, 1^st^ Faculty of Medicine, Charles University in Prague, Praha, Czech Republic; 2 Max-Delbrück-Center for Molecular Medicine, Berlin, Germany; 3 Department of Pathobiology, The Sophie Davis School of Biomedical Education, The City University of New York, New York, New York, United States of America; IGBMC/ICS, France

## Abstract

Rat hypodactyly (*hd*) mutation is characterized by abnormal spermatogenesis and sperm decapitation, limb malformation (missing digits II and III) and growth retardation. We have previously reported centrobin (*centro*some *B*RCA2-*in*teracting protein) truncation at the C-terminus in the *hd* mutant. Here, we report data from a transgenic rescue experiment carried out to determine a role of centrobin in pathogenesis of *hd*. The transgenic construct, consisting of full-length-coding cDNA linked to a ubiquitous strong promoter/enhancer combination, was inserted to chromosome 16 into a LINE repeat. No known gene is present in the vicinity of the insertion site. Transgenic centrobin was expressed in all tissues tested, including testis. Transgenic animals show normal body weight and limb morphology as well as average weight of testis and epididymis. Yet, abnormal spermatogenesis and sperm decapitation persisted in the transgenic animals. Western blotting showed the coexistence of full-length and truncated or partially degraded centrobin in sperm of the rescued transgenic animals. Immunocytochemistry showed a buildup of centrobin and ODF2 (outer dense fiber 2) at the sperm decapitation site in the *hd* mutant and rescued transgenic rats. Additional findings included bulge-like formations and thread-like focal dissociations along the sperm flagellum and the organization of multiple whorls of truncated sperm flagella in the epididymal lumen. We conclude that centrobin is essential for normal patterning of the limb autopod. Centrobin may be required for stabilizing the attachment of the sperm head to flagellum and for maintaining the structural integrity of the sperm flagellum. We postulate that the presence of truncated centrobin, coexisting with full-length centrobin, together with incorrect timing of transgenic centrobin expression may hamper the rescue of fertility in *hd* male rats.

## Introduction

About 9% of couples suffer from infertility on a global scale [1]. Male factor is a major contributing cause in about half of the cases of infertility. About half of human male infertility cases remain idiopathic despite advances in clinical diagnostics. Some of the infertility cases are thought to be of genetic origin [2]. Several genes involved in male fertility have been identified using animal models, in particular mouse targeted mutations and spontaneous mutations/transgenic models of mice and other mammals. Mutated genes can cause either standalone or syndromic male infertility [2], [3]. These mutations were shown to disrupt multiple steps of spermatogenesis and sperm function. Consequently, the identification and characterization of novel proteins involved in sperm development are key issues leading to a better understanding of presently unknown causes of male infertility.

Spermiogenesis is a complex postmeiotic phase of spermatogenesis. It involves the development of round spermatids into mature spermatids, consisting of elongated heads with an acrosome-acroplaxome complex anchored to the nucleus. The head is attached to the flagellum by the head-tail coupling apparatus (HTCA). The distal centriole of the HTCA degenerates in rodent sperm [4], [5]. The sperm flagellum consists of an axoneme surrounded by mitochondria at its head proximal segment, the middle piece, and characteristic peri-axoneme outer dense fibers along the entire length of the flagellum and a fibrous sheath in the principal piece. The annulus marks the transition from the mitochondria-containing middle piece to the principal piece of the sperm flagellum. Mature spermatids are released into the lumen of the seminiferous tubule by the process of spermiation, assisted by the apical domain of Sertoli cells, and undergo a maturation process in the epididymis defined by the acquisition of forward motility [6]–[8]. The orderly sequence of sperm development and maturation requires the timely expression, intramanchette transport (spermatids) and intraflagellar transport (spermatids and sperm) of proteins and their precise assembly and stability [9]. Several genes encoding specific proteins have been identified as essential for spermiogenesis. One of these genes is *Cntrob* (centrobin, for centrosome BRCA2 interacting protein [10]). *Cntrob* encodes a protein with a central coiled-coil region flanked by noncoiled regions at the NH_2_ and COOH termini. Centrobin is a daughter centriole protein essential for centrosome duplication and elongation, two functions depending on centrobin-tubulin interaction [11]. Centrobin regulates the attachment of the mitotic spindle to the centrosome [12], a condition essential for the stability of microtubules anchored to the kinetochore on metaphase chromosomes during separation of their chromatids. These properties are lost in centrobin-depleted cells that subsequently undergo apoptosis. When overexpressed, centrobin forms bundle-like structures [10]. The reported centrobin-microtubule association mimics a similar condition seen in spermatids and sperm, wherein centrobin associates transiently with the microtubule-containing manchette and with the centrosome and axoneme of the developing sperm flagellum.

We have previously reported the spontaneous mutation of *Cntrob* encoding truncated centrobin in rat hypodactyly (*hd*) [13]. A major defect during late spermiogenesis in the *hd* mutant is the detachment in maturing spermatids of the developing flagella from their HTCA, an event that leads to massive decapitation [13]. The HTCA can be regarded as a specialized centrosome surrounded by specific proteins, some of them presently unknown. The HTCA consists of a proximal centriole anchored to the spermatid nucleus and a distal centriole from which the microtubule-containing flagellum axoneme originates. Outer dense fibers and a fibrous sheath encircle the sperm axoneme. Basically, the structure and function of the HTCA resembles the centrosome, the putative site of the mitotic spindle. Centrobin is an addition to the catalog of proteins with a potential role in the integrity of the HTCA, a site leading to the “easily decapitated sperm syndrome” in humans [14]–[19]. In addition to the *hd* rat mutant, other rodent models also display sperm decapitation at the HTCA site [20], [21]. However, it remains to be determined the specific role of centrobin as a single protein or/and as part of protein complexes during the assembly and stability of the HTCA. This aim can facilitate the identification of subclinical forms of male infertility involving sperm head-tail fragility leading to decapitation during micromanipulation as part of in vitro reproductive assisted technologies.

To pursue further a better understanding of centrobin’s role in sperm decapitation, we attempted to rescue *hd* by transgenesis using a construct encoding full-length centrobin protein. We have found that limb malformation was completely rescued but sperm decapitation persisted in the transgenic males. Yet, we have made progress in determining additional structural and immunocytochemical parameters concerning the contribution of centrobin to sperm development.

## Materials and Methods

### Animals

All animal experiments were compliant with the Animal Protection Law of the Czech Republic (311/1997) which is in compliance with the European Community Council recommendations for the use of laboratory animals 86/609/ECC, and were approved by The Charles University Animal Care Committee. As *hd/hd* males are sterile, the strain carrying the *hd* mutation (WHD) was propagated by backcrossing heterozygous (*+/hd*) males to homozygous (*hd/hd*) females [13].

### Transgenic Construct

We previously amplified full-length wild-type centrobin (strain BN/Cub) coding sequence by RT-PCR from testis cDNA and cloned it into pEGFP-C1 [13]. We then subcloned this sequence-verified coding sequence into pBS-CX1-LEL, to generate ubiquitously expressing centrobin under the control of cytomegalovirus-enhancer/β-actin (CAGGS) promoter [22].

### Microinjection

Collecting rat zygotes, pronuclear microinjection of the construct DNA, zygote cultivation, and implantation to the pseudopregnant recipients was performed according to [22]. Since we were concerned the mutated animals may yield suboptimal results of embryo transfer, we used relatively genetically similar wild-type strain WKY/Bbb, subsequently introducing the transgene into the mutant animals by breeding.

### Production of Rescued Males

We identified single transgenic founder by PCR using primers Lip8_c5_2918F: ctcgacttccacctcctgtc; Lip8_3149R:TACAGTAGCAGGTCCTCAGCAG. PCR product of 343 bp was amplified from endogenous locus, 238 bp from transgenic construct. We bred this *+/+ Tg^+/−^* founder male (the endogenous locus with alleles “*+*” and “*hd*” for wild-type and mutant alleles respectively; “*Tg*” for the introgressed construct, allele+for presence, − for absence of the transgene), to a mutant female (*hd/hd Tg^−/−^*). Intercrossing the transgene positive hybrids (*+/hd Tg^+/−^*), we got the rescued males: *hd/hd Tg^+/−^* or *hd/hd Tg^+/+^*. Since our genotyping assay cannot distinguish transgenic homozygotes (*Tg^+/+^)* from heterozygotes (*Tg^+/−^*), we refer both groups as *Tg+*. In rescued animals, due to lack of outward phenotypic manifestation, we genotyped endogenous locus using primers: Lip8_11_139F: CTGGGAGCCACACTTAGGTC; LTR_3_F: CTGGGGCGGTACTATGCTAA; Lip8_c2_2235R: AACTCCTGTTGGTGCTGTCC. LTR_3_F-Lip8_c2_2235R yield 429 bp in mutants only (*hd/hd*), Lip8_11_139F-Lip8_c2_2235R yield 171 bp in wild-type only (*+/+*). Heterozygotes (*+/hd*) show both products.

### Transgene Insertion Site Localization

To reveal the insertion point of the transgene, we performed inverse PCR: we isolated DNA from a tg+ animal, cut with restriction endonuclease Hin1II (Fermentas, Vilnius, Lithuania) ligated to form circular DNA, amplified with primers CMVie_F: CAAGTACGCCCCCTATTGAC; CMVie_R: GCCAAGTGGGCAGTTTACC; and sequenced the product using Sanger sequencing with BigDye chemistry (Applied Biosystems, Foster City, California). Primers designed to amplify the junctions between chromosome 16 and transgene construct were as follows: Primers localized to chromosome 16 were chr16_44M_F: TTTGCGAGGGACCTAAAATG and chr16_44M_R: GCTGTGCGAATTGATCAGAA; primers in the construct were _tgF: CCAATTCGCCCTATAGTGAGTCGT and _tgR = CMVie_R: GCCAAGTGGGCAGTTTACC.

### Sperm Counts

We killed adult (3 months old) males and dissected cauda epididymis of both sides, placed them into 5 ml of pre-warmed PBS, cut several times with scissors and incubated for 10 min at 37°C. We counted an aliquot in Bürker hemocytometer. If needed, suspensions were further diluted before counting. More than 100 sperm were counted in each animal. Numbers were expressed as total amount of sperm yielded from one male.

### RNA Extraction and RT-PCR

**Figure 1:**
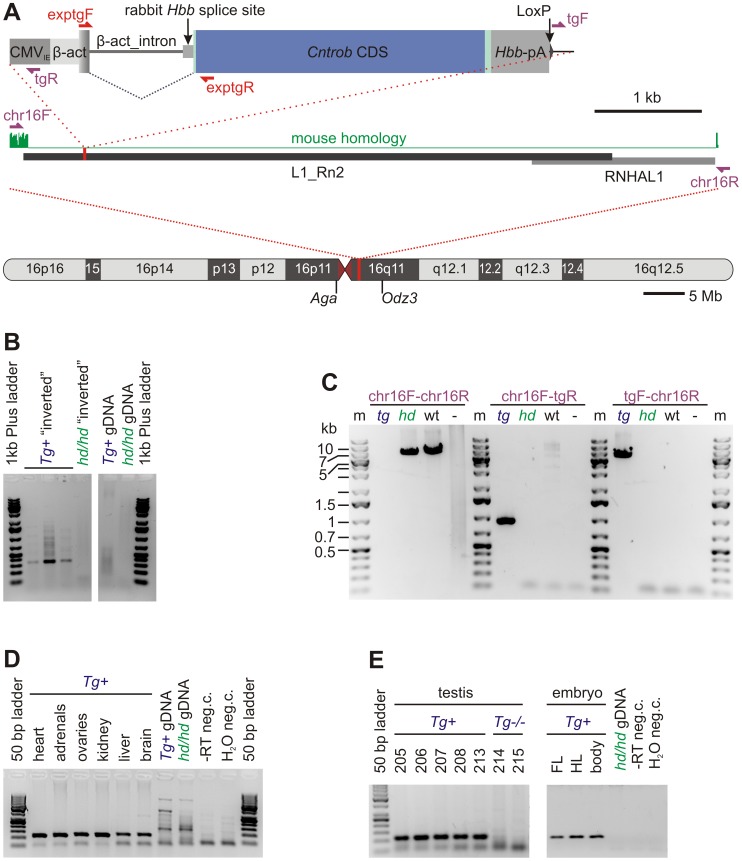
Transgenic construct, its localization and expression. **A** Top: Transgenic construct contains CMV_IE_ enhancer, chicken β-actin promoter together with first noncoding exon and intron with rabbit β-globin acceptor splice site, coding sequence and rabbit β-globin 3’UTR and polyA signal. Violet arrows represent PCR primers used to amplify the insertion sites (see **B**). Red arrows depict primers used to assess expression of the transgene (see **D** and **E**). **A** Middle: structure of the insertion site region showed in detail (genome assembly Rn3.4, chr16∶43998593–44005224). Green: homology with the mouse (above zero in the nonrepetitive flanking sequence), grey rectangles: repetitive sequences; the insertion site is red. **A** Bottom: Rat chromosome 16. Red line: insertion site; purple: centromere. The approximate positions of the genes flanking the transgene are shown. **B:** Inversion PCR amplifying a fragment of chromosome 16 only in the transgenic animals. **C:** Long range PCR confirming insertion of the transgene to chromosome 16. Expected product sizes were (for wild-type and transgenic respectively): chr16F-chr16R 6632 bp and 11898 bp (impossible to be amplified with the PCR system employed); chr16F-tgR 944 bp (only transgenic); tgF-chr16R 6080 bp (only transgenic). **D**: Expression of the transgene in somatic tissues by RT-PCR. **E:** Expression of the transgene in testis by RT-PCR. *hd/hd* or *hd* = hypodactylous mutants, transgene negative; *Tg* (*Tg+*) = transgene positive; wt = wild-type; “*−*“ in **C**: water negative control; “−” RT in **D** and **E**, reverse transcriptase dropout negative control. Numbers of individual animals according to our internal system for animal identification are given in panel **E**.

We extracted RNA from testis and other organs using Trizol (Invitrogen, Carlsbad, California) and isopropanol precipitation. For extraction of sperm RNA, we homogenized sperm isolated from single cauda/caput epididymis (for wild-type and rescued animals) or from both sides of three males (non-rescued mutants) in Trizol. After the phase separation, we isolated RNA from the aqueous phase using RNA Plus Micro Kit (Qiagen, Hilden, Germany) according to manufacturer’s instructions. We reversely transcribed the total RNA using Superscript III (Invitrogen, Carlsbad, California) and amplified using primers _exp_tgF: CTGACTGACCGCGTTACTCC; _exp_tgR: GCTGTAGCCATGTGCAGAGA; to assess expression of the transgenic construct. Note that the primer combination takes advantage from the intron introduced in the construct, which eliminates interference with endogenous, as well as possible contamination of RNA samples with genomic DNA of the transgenic animals (see **Figure 1** **,A, D, E**). Primers flanking the junction between exons 10 and 11 were used for amplification of both endogenous and transgenic cDNA distinguishing wild-type (both endogenous and transgenic) and *hd* alleles [13].

### Protein Extraction from Sperm and Western Blotting

For sperm protein isolation, we took the organic phase of Trizol left after RNA isolation and proceeded to extract proteins according to manufacturer’s instructions, except that we added DTT to final concentration of 50 mM at the beginning. We also used DTT at this concentration in the resuspension buffer, containing 1% SDS. After standard SDS-PAGE, we detected centrobin using affinity-purified rabbit polyclonal antibodies against N-terminal or C-terminal domains of centrobin. To produce the antibodies, we amplified fragments of *Cntrob* from testicular cDNA, cloned into pET-15b (EMD Millipore Biosciences, Billerica, MA) overexpressed the His-tagged recombinant proteins in Rosetta(DE3)pLysS cells (EMD Millipore Biosciences, Billerica, MA) and purified soluble proteins using Ni-NTA resin (Qiagen, Hilden, Germany). We then exchanged the buffer to phosphate-buffered saline using PD-10 desalting columns and immunized rabbits with 100 µg/kg recombinant protein in complete (first dose) or incomplete (subsequent doses) Freund’s adjuvant (Sigma-Aldrich, St. Louis, MO). We applied up to six doses monitoring antibody response by dot-blot. We purified the collected serum using the recombinant proteins covalently coupled to NHS-Activated Sepharose 4 Fast Flow (GE Healthcare Bio-Sciences, Little Chalfont, United Kingdom). Elution buffer was glycine pH 3.0, instantly neutralized by addition of 1/20 volume of 1M Tris pH 9.0. After concentration and exchange to Tris-buffered saline using Vivaspin columns (Vivaproducts, Littleton, MA) and adjustment to 50% glycerol, the antibodies were stored at −20°C. Western blotting was performed with anti-centrobin antibodies at final concentration 0.5 µg/ml, secondary HRP-conjugated antibody (GE Healthcare Bio-Sciences, Little Chalfont, United Kingdom), and signal was detected using ECL Advance or ECL Prime chemiluminiscent detection kit (GE Healthcare Bio-Sciences, Little Chalfont, United Kingdom). Control monoclonal β-actin antibody was purchased from Abgent (San Diego, California).

### Immunofluorescence and Electron Microscopy

All procedures, including processing, sectioning and staining of plastic embedded tissues for histological examination, preparation of spermatogenic cells from isolated seminiferous tubules and sample preparation for immunofluorescence and electron microscopy were previously described. [13], [23]. ODF2 polyclonal antiserum was produced and characterized as described [24]. Affinity purified anti-centrobin serum detecting the N-terminal domain of centrobin was used at a working dilution of 1∶200. Affinity purified anti-ODF2 serum was used at a working dilution of 1∶250.

## Results

### Transgenesis

The construct used for transgenesis caries the full-length wild-type centrobin coding sequence (GenBank accession number EF532342.1) under the control of the CMV immediate-early enhancer and the chicken β-actin promoter. The construct also contains an intron from the chicken β-actin gene combined with a rabbit acceptor splice site, and rabbit hemoglobin polyA signal (**Figure 1** **,A**). This design should support ubiquitous expression [22].

We microinjected this construct into zygotes of the WKY/Bbb inbred strain, identified a single founder showing successful integration of the transgene, and bred the founder with the mutant *hd/hd* females to get the rescued animals.

We assessed chromosomal localization of the transgene using inverse PCR (**Figure 1** **,B**). Sequencing of the amplified fragment revealed LINE type of repetitive sequence, which can be nevertheless mapped to rat chromosome 16 with high probability (the only 100% sequence identity hit by BLAT [25] on the rat reference genome v3.4). Subsequent PCR amplification of both 5′ and 3′ joints using primers anchored to the unique sequence flanking the LINE repeat confirmed localization of the transgene at 16q11 (**Figure 1** **,C**).

**Figure 2:**
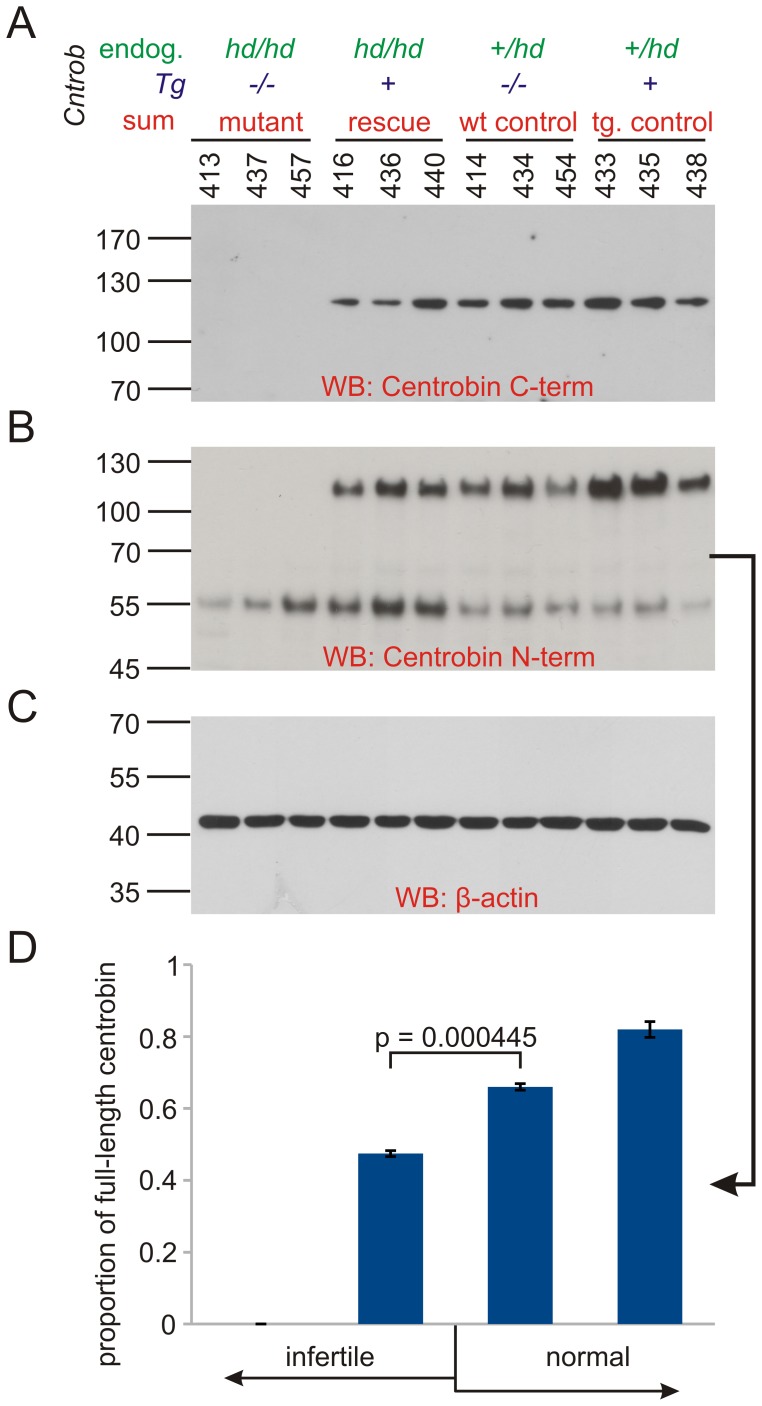
Detection of centrobin in testis by Western blotting. **A**: Western blot using centrobin C-terminal antibody that recognizes both endogenous and transgenic wild-type centrobin. *hd*-allele specific truncated form cannot be detected. Lack of centrobin in mutants is supplemented to approximately normal level in the rescued males. **B**: Centrobin N-terminal antibody confirms presence of wild-type protein and the *hd*-specific truncated protein (lower amount in heterozygote controls). **C**: We used β-actin as a loading control. **D**: Relative amount of full-length centrobin as determined from densitometry of the Western blot in B. The proportion is different among groups with full-length centrobin (ANOVA p = 10^−5^), post-hoc comparison by Tukey’s test shows significant difference between the adjacent infertile and fertile groups (p = 4.45 10^−4^). Numbers labeling the lanes identify individual animals.

The transgenic construct was expressed in all organs tested (**Figure 1** **,D**) including testes and limb buds (**Figure 1** **,E**) as shown by RT-PCR and also by western blot (**Figure 2**).

### Rescue of Limb Malformation

**Figure 3:**
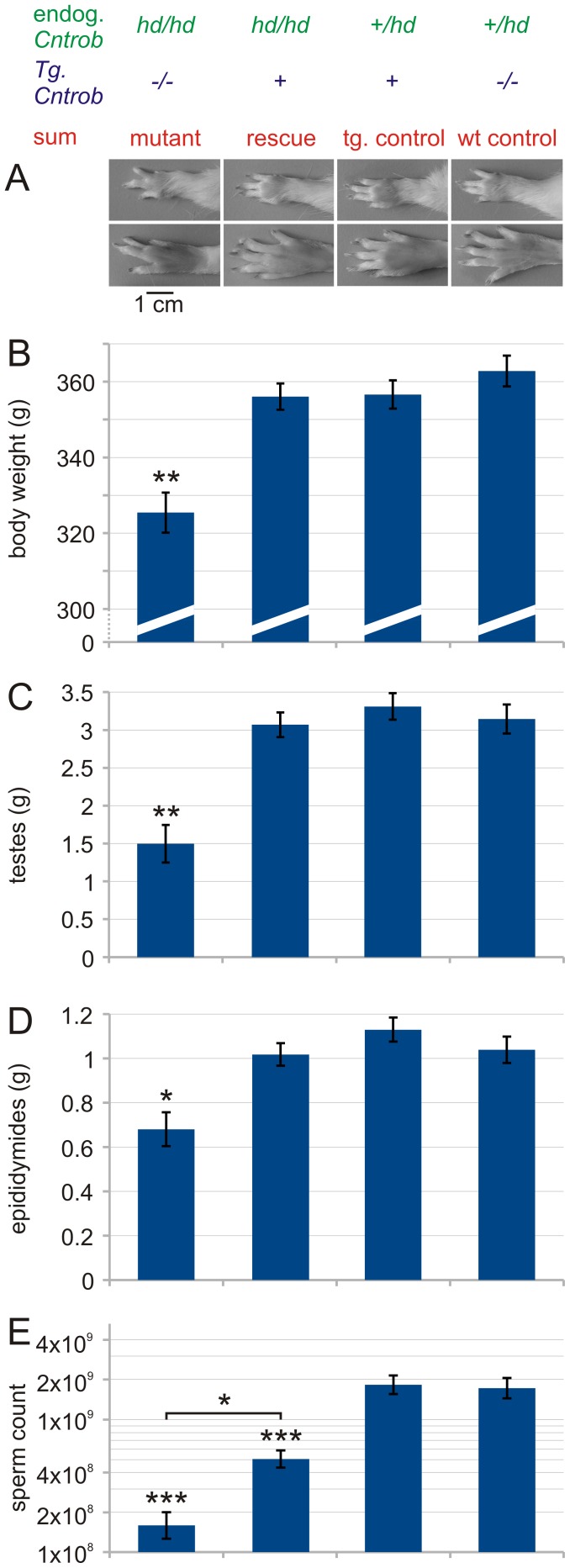
Limb phenotype, growth retardation rescue and improvement of reproductive phenotype. **A**: Autopods of mutants compared to transgenic rescue and controls. Left forelimb (top) and hind limb (bottom). Anterior to posterior axis from top to bottom. Normal hind limb has 5 toes, normal forelimb has 4 fingers and a rudimentary thumb. Note missing digit II and hypoplastic digit III in mutants only. **B**: Body weight. Transgenic rescue rats are indistinguishable from controls, mutants are significantly smaller (post-hoc P = 0.0039 in comparison to rescued males). **C**: Testis weight (both testes together). Transgenic rescued rats are indistinguishable from controls, mutants are significantly smaller (post-hoc P = 0.0018 in comparison to rescued males). **D**: Epididymis weight (both epididymides) Transgenic rescue rats are indistinguishable from controls, mutants are significantly smaller (post-hoc P = 0.0297 in comparison to rescued males). **E**: Sperm count (cauda epididymidis of both sides). Rescued males have more than 3 times more spermatozoa compared to mutants (post-hoc P = 0.00114). However, control males have at least 4 times more spermatozoa compared to rescued males (post hoc P = 0.000816 for rescued males compared to wild-type males). Mutants n = 3, rescued n = 7, transgenic controls n = 6, nontransgenic (wild-type = WT) controls n = 5. One-way ANOVA, with post hoc Tukey test for unequal N.

Rat hypodactyly shows, aside of male infertility, a characteristic limb malformation [26]. The limbs of rescued males (*hd/hd Tg+*) cannot be distinguished from the normal limbs at the gross anatomy level while their littermates (*hd/hd Tg^−/−^*) possess the typical reduction of digit II and III (**Figure 3** **,A**). We conclude that the limb malformation is completely rescued.

### Rescue of Normal Growth

Another pathological manifestation of the *hd/hd* mutants is a slight growth retardation resulting in decreased body weight of adults. This phenotype is also rescued by the centrobin transgene (**Fig. 3B**).

### Testicular and Epididymal Weight

*hd/hd* mutants have much smaller testes when compared to wild-type controls. The testicular and epididymal weights of rescued animals were comparable to controls (**Figure 3C,D**). Cauda epididymides of the rescued males contained significantly more decapitated sperm remnants but less whorls of flagella than in the *hd* mutant. Yet, control males had significantly more sperm in cauda epididymidis (**Figure 3E**).

### Sperm Morphology Remains Abnormal Despite the Expression of Full-length Centrobin

**Figure 4:**
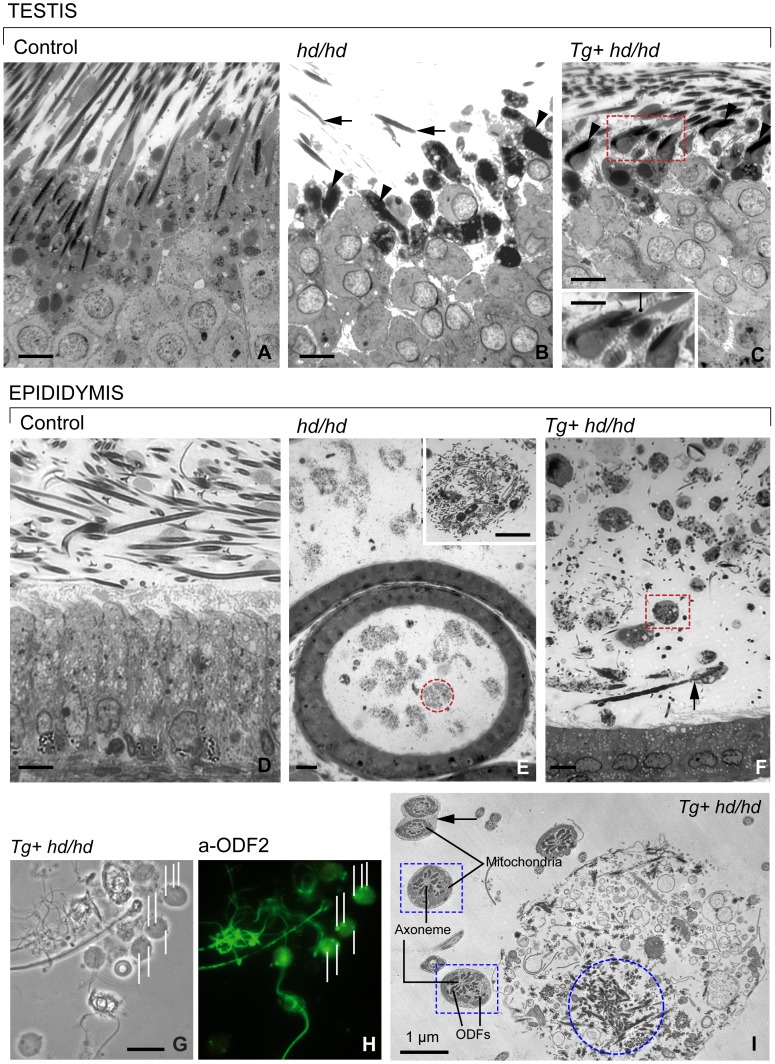
Characteristics of spermiation and sperm maturation in control, *hd/hd* mutant and rescued *Tg+ hd/hd* rats. **A:** Histologic section of a control seminiferous epithelium showing the release of mature spermatids during stage IX of spermatogenesis. **B:** Histologic section of a *hd/hd* mutant displaying abnormally shaped heads (**arrowheads**) and severed flagella (**arrows**) of mature spermatids seen in a similar spermatogenic stage IX. Note that round spermatids display normal structure. **C:** Histologic section of a rescued *Tg+ hd/hd* rat showing heads (**arrowheads**) separated from the flagella seen in a similar spermatogenic stage IX. The **inset** illustrates a thin cytoplasmic bridge (**pointer**) connecting the spermatid head to its developing flagellum. The number of decapitated mature spermatids released at spermiation is larger in the rescued rat as compared to the mutant. Round spermatids display normal features. **D:** Histologic section of the tail region of the epididymis of a control rat showing well-developed sperm in the lumen and the epididymal epithelium with normal characteristics. **E:** In the *hd/hd* mutant, the epididymal lumen contains multiple compact whorls (**dashed circle**) each consisting of entangled flagella seen in the **inset** at higher magnification. The height of epididymal epithelium is reduced. F: In the rescued *Tg+ hd/hd* rat epididymis, flagellar whorls coexist with numerous spherical bodies (**dashed box**). The **arrow** indicates a decapitated sperm. The height of epididymal epithelium is reduced. **G–H:** Phase contrast microscopy (panel **G**) and immunofluorescent localization of ODF2 (panel **H**) in decapitated sperm and spherical bodies harvested from the epididymal cauda of a rescued *Tg+ hd/hd* rat. The **pointers** in panel **H** indicate immunoreactive ODF2. **I:** Electron microscopy of a spherical body and sperm flagella in the epididymal lumen of a *Tg+ hd/hd* rat. The **dashed circle** indicates aggregates of outer dense fibers in the spherical body equivalent to those seen in panels **G** and **H**. The **dashed boxes** indicate cross-sections of sperm flagella (middle piece) each with an intact axoneme and surrounded by fragmented outer dense fibers (**ODFs**). The **arrow** indicates two fused sperm flagella (principal piece). Scale bar in all panels and inset is 5 µm.

Our first step was to distinguish differences in the structure of the seminiferous epithelium in normal, *hd/hd* mutant and *Tg+ hd/hd* rats. **Figure 4** **,A–C** compares spermiation during stage IX of spermatogenesis in the three rat models. In control rats, spermiation proceeds with the expected release of well-developed mature spermatids into the seminiferous tubular lumen. In contrast, spermiation in the *hd/hd* mutant and *Tg+ hd/hd* rats consists in the release of partially or fully decapitated mature spermatids. A significant difference is the relative abundance of releasing mature spermatids in *Tg+ hd/hd* rats when compared to the *hd/hd* mutant. The caput, corpus and cauda of the epididymal duct were examined in normal, *hd/hd* mutant and *Tg+ hd/hd* rats (**Figure 4** **, D–F**). Contrasting with the expected presence of abundant normal sperm in the lumen of cauda epididymidis (**Figure 4** **, D**) was the profusion of whorls of decapitated sperm flagella in the *hd/hd* mutant (**Figure 4** **, E** and **inset**) and spherical bodies in the epididymal cauda of *Tg+ hd/hd* rats (**Figure 4** **,F**). An immunocytochemical analysis of specimens collected from the epididymal cauda of *Tg+ hd/hd* rats indicates the presence of ODF2 immunoreactive sites in the spherical bodies (**Figure 4** **,G,H**). Electron microscopy confirmed the presence of bundles of outer dense fibers (ODFs) in the spherical bodies as well as the fragmentation and detachment of ODFs from the microtubule-containing axoneme of the flagella (**Figure 4** **,I**). We concluded that the multiple whorls seen in the epididymal lumen resulted from the aggregation of underdeveloped decapitated flagella and that the spherical bodies originated from the fragmentation of flagella. Similar histological studies of *Tg+ +/hd* rats revealed normal structural features comparable to those seen in control rats (data not shown).

### Mislocalisation of Centrobin and ODF2 in Epididymal Sperm of Transgenic Rats

**Figure 5:**
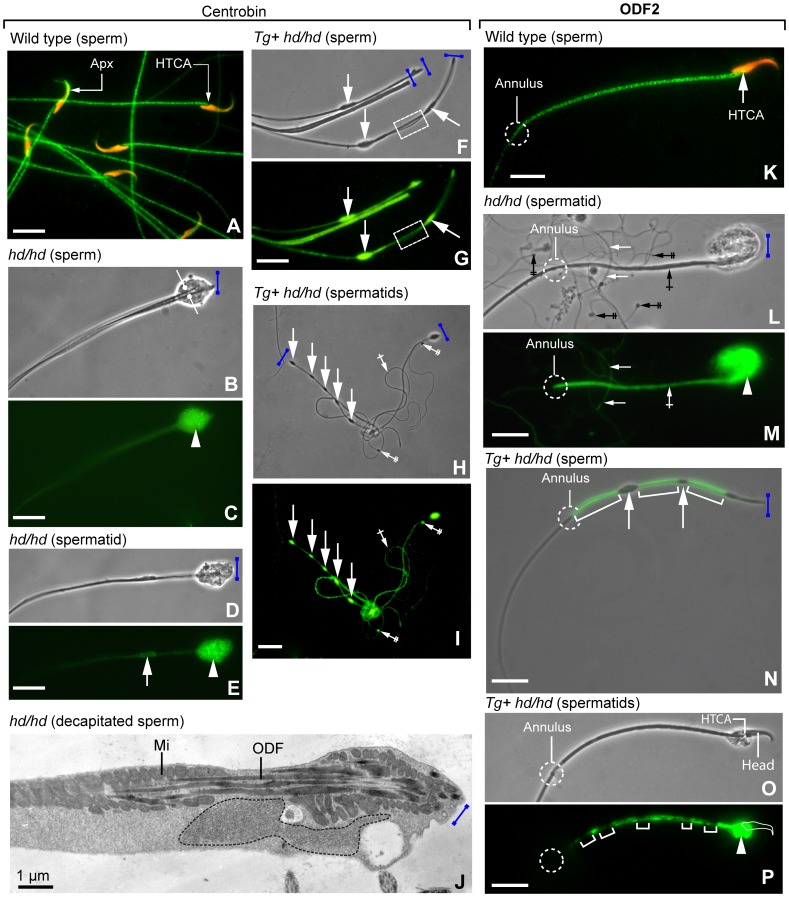
Immunofluorescence localization of centrobin (A, C, E, G and I; N-terminal antibody) and ODF2 (K, M, O and Q) in spermatids and/or epididymal sperm from control, *hd/hd* mutant and rescued *Tg+ hd/hd* rats. Panels B, D, F, H, L and P are phase-contrast microscopy. Panel **O** is a phase-contrast microscopy-immunofluorescence merge. The **blue bar** indicates the decapitation region. **A:** Centrobin localization in wild-type control epididymal sperm. The location of the immunoreactive acroplaxome (**Apx**) and head-tail coupling apparatus (**HTCA**) is indicated. Note the regular immunoreactive centrobin banding pattern along the flagellum. **B–C**: Decapitated sperm with fused flagella (**opposing arrows**). Centrobin immunoreactivity predominates at the HTCA region (**arrowhead**). **D–E**: Decapitated spermatid. The **arrowhead** indicates substantial centrobin-containing material at the HTCA region. The **arrow** points to a developing bulge of the flagellum. **F–G**: The **arrows** denotes the presence of centrobin in the flagellar bulges. The **dashed box** indicates a thread-like dispersion at the inter-bulge linker. **H–I**: The **arrows** show the linear arrangement of centrobin-containing bulges along the spermatid flagellum. The **single-crossed arrow** identifies immunoreactive spermatid flagella of reduced diameter. The **double-crossed arrows** point to the centrobin-stained severed end of the thin, also immunoreactive, decapitated spermatid flagella. **J**: Transmission electron microscopy of a decapitated sperm. The **dotted line** indicates a large deposit of proteinaceous material at the decapitated end. The material is presumably equivalent to the accumulation of centrobin and ODF2 (and other proteins) detected by immunocytochemistry. The localization of outer dense fibers (**ODF**) and mitochondria (**Mi**) is indicated. **K**: Wild type control epididymal sperm stained with ODF2 antibody. The location of the HTCA and annulus is indicated. **L–M**: The **arrowhead** indicates a large deposit of ODF2 at the HTCA region and extending along the middle piece of the flagellum (**single-crossed arrow**) up to the annulus. The **double-crossed arrows** point to densities at the decapitated end of several developing spermatid flagella. The **arrows** denote less intense ODF2 immunoreactive spermatid thin flagella contrasting with the more intense staining of the thicker flagellum (**single-crossed arrow**) present in the field. **N:** Merged phase-contrast-fluorescence microscopy images displaying ODF2 immunoreactivity along segments (**brackets**) linking non-immunoreactive bulges (**arrows**) of a decapitated sperm flagellum. ODF2 immunoreactivity extends up to the annulus. **O–P**: Mature spermatids with a persistent attached head can be seen. A significant ODF2 deposit is visualized at the HTCA region (**arrowhead**). The **brackets** indicate a less intense staining of ODF2 in regions presumed to become bulges. Scale bar in phase-contrast and fluorescence microscopy panels: 10 µm.

Sperm of the rescued *Tg+ hd/hd* rats were immotile (data not shown). Despite mating of multiple rescued males with fertile females for several months, no offspring was observed. This outcome was in accordance with the structural testicular and epididymal data. Our next step was to compare the localization of centrobin and ODF2 in mature spermatids and sperm in wild-type rat, *hd/hd* mutant and *Tg+ hd/hd* rats. We previously determined that decapitation in *hd/hd* rats takes place upon completion of spermiogenesis [13]. Here we wanted to examine the relationship between centrobin and ODF2, a specific marker of outer dense fibers of the flagellum and also a component, together with centrobin, of the HTCA [13], [24]. **Figure 5** **,A–J** illustrates centrobin immunoreactive patterns in epididymal sperm and mature spermatids of wild-type, *hd/hd* mutant and rescued *Tg+ hd/hd* rats visualized with an antiserum detecting the N-terminal domain of centrobin. In normal sperm, centrobin is visualized in the acroplaxome region of the sperm head, the HTCA and along the flagellum (**Figure 5** **,A**). In the *hd/hd* mutant, decapitated sperm (**Figure 5** **,B,C**) and decapitated mature spermatids (**Figure 5** **,D,E**) display a centrobin-containing mass at the HTCA region. Three significant characteristics were noted in epididymal sperm (**Figure 5** **,F,G**) and mature spermatids (**Figure 5** **,H,I**) from rescued *Tg+ hd/hd* rats: (1) the distribution of centrobin immunoreactive sites along the flagellum extended distally beyond the HTCA region into the flagellum. (2) An intermittent scattering of centrobin immunoreactive bulges was seen along the flagellum. (3) Tail components dissociated into thin threads at discrete focal points between bulges. We concluded that, although centrobin extended beyond the HTCA region in *Tg+ hd/hd* spermatid and sperm flagella, centrobin immunoreactivity was preferential at the bulging regions. Furthermore, a thread-like dissociation of flagellar segments was restricted to those devoid of significant centrobin immunoreactivity (see the **dashed box** in **Figure 5** **,F,G**). **Figure 5** **,J** illustrates the partial dissociation of ODFs, the mislocalization of mitochondria and accumulation of proteinaceous material in a decapitated epididymal sperm from the *hd/hd* mutant resolved by electron microscopy. Similar changes were seen in rescued *Tg+ hd/hd* rats (data not shown).

An antibody to ODF2 reveals specific immunoreactivity at the HTCA region, along the middle piece of the flagellum, and with somewhat decreased intensity along the principal piece of a wild-type sperm (**Figure 5** **,K**). In spermatids from the *hd/hd* mutant, ODF2 aggregates at the decapitated end of the developing spermatid flagellum with moderate immunoreactivity extending toward the annulus region (**Figure 5** **,L,M**). Relatively thin developing flagella, caped at one end by a severed HTCA, can be seen forming an entangled network. A significant observation was the lack of ODF2 immunoreactivity in the bulging flagellar region of late spermatids and epididymal sperm from rescued *Tg+ hd/hd* rats (**Figure 5** **,O**). Immunoreactive “silent” flagellar segments can be seen in a late spermatid (that still retains its attached head) to an abnormal aggregate of immunoreactive ODF2 at the HTCA region (**Figure 5** **,P,Q**). We concluded that the centrobin-rich bulges may represent a storage area of a centrobin excess in the sperm flagellum of rescued *Tg+ hd/hd* rats. This condition was not paralleled by an equivalent deposit of ODF2. In rescued late spermatids, ODF2 distribution was relatively discontinuous along the developing flagellum, while most of the bulk of ODF2 was restricted, together with centrobin, to the HTCA region (compare **Figure 5** **,D,E** and **Figure 5** **,P,Q**).

### Higher Levels of Centrobin Transcripts and Protein are Present in Sperm of Rescued *Tg+ hd/hd* Rats

**Figure 6:**
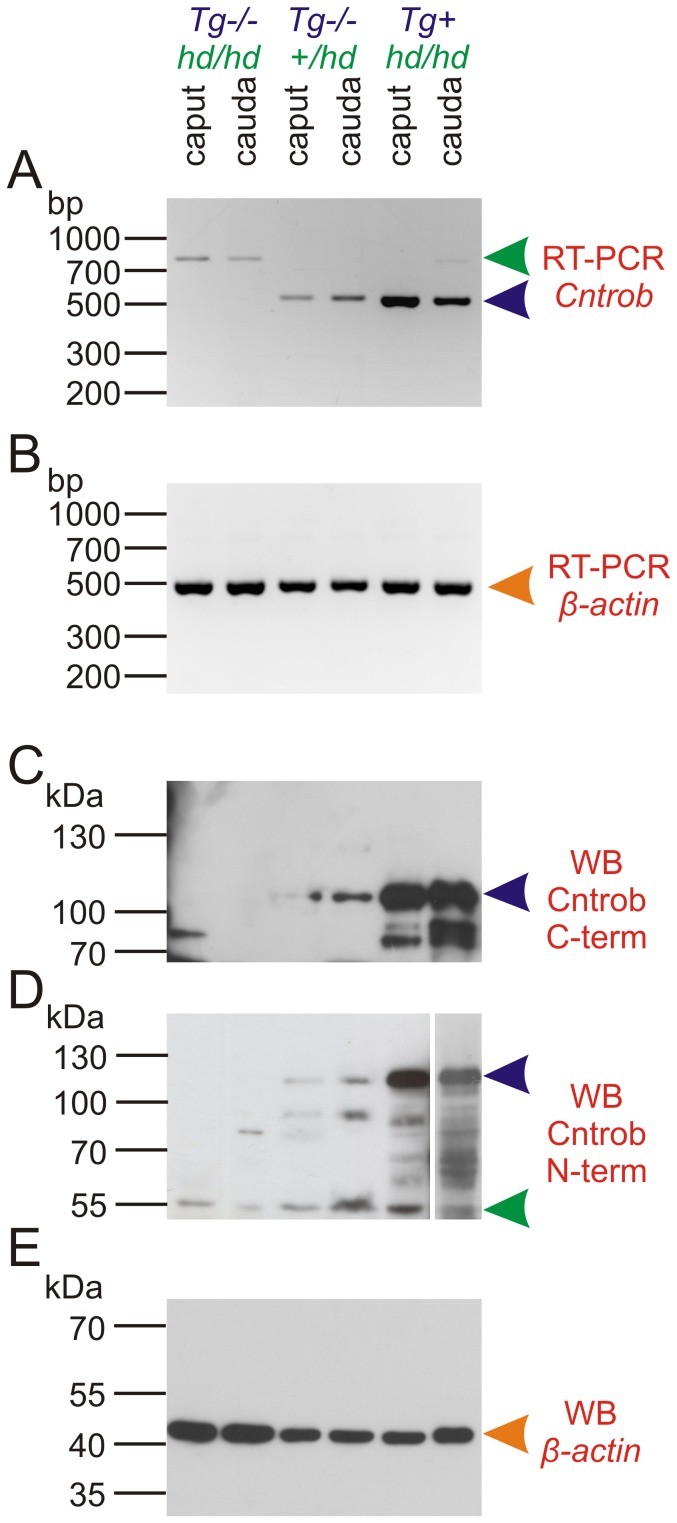
Centrobin expression in epididymal sperm. **A**: RT-PCR with primers flanking the junction between exon 10 and 11. Mutants show products with higher molecular weight due to the insertion of an endogenous retrovirus [33]. (**green arrowhead**). Wild-type product (**blue arrowhead**) can be amplified both from endogenous locus transcripts as well as from the transgenic transcripts. The “mutant” product can be also seen in the rescued males, although during PCR, the product can be masked by preferential amplification of the smaller molecular weight amplicon coming from the transgene. **B**: β-actin is shown as endogenous control. Robust expression of centrobin can be seen in sperm both from caput (less mature) and cauda (more mature) epididymis. **C**: Western blot with an antibody against C-terminal part of centrobin. There is no signal in mutants because they lack the C-terminal portion of centrobin. Note a lower molecular weight species of centrobin from from caput and cauda epididymidis sperm of rescued rats that may correspond to proteolytic processing or degradation. This can be seen in the wild-type sperm too with higher exposition time (not shown). **D**: Western blot with an N-terminal-specific antibody (middle panel) shows similar results as seen with the C-terminal antibody, 55 kDa mutant truncated protein is also identifiable (**green arrowhead**). Note: the last lane had to be developed separately due to excess signal. **E**: β-actin served as the loading control.

*Cntrob* transcripts were expressed in sperm isolated both from caput and cauda epididymidis. It was also possible to distinguish wild-type and mutant transcripts (**Figure 6** **,A,B**). In sperm of the rescued *Tg+ hd/hd*, the expression of transgenic centrobin can be clearly observed at a significantly higher level in comparison to both non-transgenic controls and mutants. Transcript abundance was reflected by protein abundance measured by western blotting (**Figure 6** **,C–E**). We concluded that differences in the level of centrobin transcripts and protein correlated with a more extensive distribution of centrobin in sperm of rescued *Tg+ hd/hd* rats as compared to the *hd/hd* mutant. **Figure 6** **,C** shows a protein band with a lower molecular mass which appears to represent truncated centrobin (100–70 kDa range, different from the cca 55 kDa protein from *hd/hd* males) perhaps representing proteolytic cleavage, coexisting with full-length centrobin (110 kDa) over-expressed in rescued rats. In fact, centriolar degeneration or reduction normally occurs when rat sperm reach the epididymis [4], [5]. It is likely that a centrobin proteolytic cleavage reflects in part the centrosomal involution process that may be more prominent in sperm of the *hd/hd* mutant.

## Discussion

*Cntrob* mutation in the rat *hypodactyly* (*hd*) was discovered using the positional cloning approach. The non-recombinant region in the case of *hd* was 464 kb and contained 16 genes. All the evidence pointed to truncated centrobin protein encoded by the mutated *Cntrob* gene in the *hd* mutant rat as causative of skeletal abnormalities and male infertility [13]. In this work, we attempted the transgenic rescue of the *hd* mutant rat, a golden standard for validating the role of *Cntrob* in skeletal and infertility pathogenesis. As we have shown previously, the characteristic limb phenotype of *hd* rats is comprised of reduction of distal parts of digits II and III. On molecular level, limb buds of *hd* rats show deficiency of chondrogenic condensation of distal parts of digital rays II and III, as shown by Sox9 expression. The data presented here show that the transgenic rescue fully corrected the autopod reduction phenotype. (**Figure 3A**). We conclude that *Cntrob* mutation is responsible for skeletal alterations of the *hd* mutant rat. Therefore normal function of centrobin is essential for autopod development. Growth retardation was also fully corrected (**Figure 3B**). This finding supports previous evidence from cellular studies, suggesting that centrobin role in centriole duplication has impact on cell proliferation rate [10], [11].

Male infertility was persistent in transgenic *hd* mutant males. Testicular and epididymal gross anatomy of the rescued rats did improve to wild-type level (**Figure 3C,D**). However, the sperm count, albeit meaningfully higher compared to mutants, was considerably lower compared to wild-type controls. Despite this relatively increased amount of sperm, the key finding impacting on the fertility of the rescued males was the persistent and substantial sperm decapitation. We could not detect significant differences in the decapitation pattern of rescued and *hd* mutant sperm. Yet, we observed structural changes as well as differences in the immunoreactive pattern of centrobin and ODF2 along the sperm flagellum pointing to a role of centrobin in stabilizing the association of ODFs to the axoneme. A significant structural finding was the series of sperm flagellum bulges housing centrobin but not ODF2. ODF2 was restricted to the inter-bulge connecting segments, which showed a tendency to break up into thread-like formations (see **Figure 5F,G**). It is likely that the bulges along the sperm flagellum of the rescued *Tg+ hd/hd* rats develop to house excess full-length centrobin coexisting with endogenous truncated centrobin and other proteins. A high concentration of centrobin in the bulges may disrupt the intraflagellar transport of centrobin and other proteins to the required assembly sites. As a result, segments of the flagellum become less stable. Examples of these proposed mechanism could be the distinct thread-like dissociation sites (see **Figure 5F,G** **),** the fragmentation and dispersion of outer dense fibers (see **dashed boxes** in **Figure 4I**), and the release into the epididymal lumen of spherical bodies containing dissociated outer dense fibers (see **Figure 4** **G–I**).

It is also possible that centrobin may stabilize the association of outer dense fibers to the microtubule-containing axoneme. The sperm head-tail attachment site consists of centriolar microtubules and pericentriolar proteins developing the HTCA. ODF2 and centrobin are components of the HTCA [13], [24]. The presence of large aggregates of centrobin and ODF2 at the HTCA site (see **centrobin** in **Figure 5** **,D,E** and **ODF2** in **Figure 5** **, L,M**) suggests a disruption in the transport of these two proteins along the developing flagellum. In fact, bundles of thin flagella seen in testis (see **Figure 5** **,H,I** and **Figure 5** **,L,M**) and prominent whorls of flagella seen in epididymis (**Figure 4** **,E,F and** **Figure 4** **,I**) indicate that decapitation occurs to a large extend before completion of flagellar development. Decapitated flagella find it easy to form multiple whorls and undergo a degradation process in the epididymal environment resulting in the formation of multiple spherical bodies.

Recently two targeted gene inactivation experiments were reported to result in sperm head decapitation, Odf1 [21] and Oaz3 (OAZ-t) [20]. Odf1 is a major component of the outer dense fibers. Odf1^−/−^ mice show isolated deficiency of morphogenesis of elongated spermatids. In addition to deficiency of, the flagella show disturbed mitochondrial sheath and dislodged outer dense fibers. Interestingly, detached flagella retain some motility. *hd* mutants as well as *Cntrob* transgenes similarly to Odf1^−/−^ display outer dense fiber disorganization (thread-like dissociation of ODF2 positive material). Also limited motility of the detached flagella can be observed in fresh preparations of caput epididymidis sperm (unpublished data) indicating (limited) functionality of detached tails. On the other hand, distinct coiling of the flagellum typical for Odf1−/− sperm was not observed neither in *hd* nor in *Cntrob* transgenic rats, that instead form whorls of multiple flagella. OAZ3 is an inhibitor of ornithine decarboxylase likely regulating local content of polyamines in the developing sperm. Oaz3 deficient mice show easy separation of heads from flagella. There are few points of interest – ultrastructure of the flagellum and HTCA is well preserved (in contrast to both Odf1−/− and hd), detachment is well defined to occur between basal plate of the nuclear envelope and capitulum, nondisrupted sperm can be retrieved from epididymis and lose tails upon incubation, these tails display “mighty motility” up to 15 hours. These comparisons suggest a model, where centrobin participates with Odf1 in building the outer dense fibers, attaching mitochondria and making structurally tough HTCA. Centrobin perhaps is not part of the ODFs themselves as suggested by its relation with Odf2 protein in centrobin transgenes. Polyamines probably act later in final stabilization of the structures of HTCA although the mechanism is speculative. Supportive evidence comes from a study showing that primary amines disrupt head to flagellum connection [27].

We are at present unable to demonstrate that *Cntrob* mutation is a direct determinant of decapitation leading to male infertility in *hd* rats. It is possible that, in addition to *Cntrob*, the mutation of another gene may be responsible for sperm decapitation in *hd* rats, or that a negative effect determined by truncated centrobin is not fully compensated or overridden by the coexisting expression of full-length centrobin.

First possibility of an additional contributing gene would point to one of the remaining 15 genes in the *hd* non-recombinant region as a cause of rat *hd*. However, 4 of the 15 genes are not expressed in the testis and the rest do not contain any mutation in the coding sequence nor any mutation affecting splicing. Also RT-PCR did not indicate any substantial change in the expression of these genes. All available evidence thus still points to the *Cntrob* gene as responsible for male infertility of *hd* mutant [13].

The possibility of interference of the mutant truncated centrobin with full-length transgenic centrobin is not compliant with the fact that heterozygotes *+/hd* show normal fertility and are indistinguishable from wild-type (*+/+*) males suggesting hd allele is operating more like a loss-of-function mutation. Substantial level of centrobin observed in the testis as well as in the sperm of the transgenic males makes this hypothesis unlikely. This finding also excludes epigenetic transgene silencing as the cause of the persisting infertility phenotype, even though the transgene is localized inside a LINE element that may be selectively silenced [28], [29]. On the other hand deficiency in Piwi/piRNA mediated LINE silencing lead to male infertility [30]–[32], and we may speculate what would happen if the transgenic construct were able to rescue the LINE element at the site of transgene insertion from the silencing process. However; this LINE element (belonging to family L1) is incomplete (lacks 5′ 1.2 kb and 3′ 1.5 kb), likely inactive, and thus not direct target of silencing. Therefore, the non-native promoter used is probably deficient in the normal temporal regulation of centrobin expression which may result in centrobin protein expression at the wrong time and possibly in the wrong amount during spermatogenesis, in other words, we can expect dosage sensitivity of centrobin as part of functional protein complexes that would be disrupted either in absence or excess of centrobin. This hypothesis is supported by the finding of impaired localization of centrobin in transgenic rescued rats. An intriguing possibility would be requirement of a different splicing variant of centrobin in spermatids in contrast to somatic cells. However, RT-PCR data do not indicate coexistence of splicing transcript variants encoding full-length and truncated centrobin.

Further analysis of the mechanism of sperm decapitation and flagella abnormalities is hindered at present by a lack of data concerning centrobin-interacting protein partners during sperm development. It is known that α-tubulin is a centrobin-interacting partner in the centriole and that centrobin-tubulin interaction is necessary for centriole elongation [11]. Since HTCA contains a centriole pair, centrobin may be recruited to HTCA via its tubulin interaction. Furthermore, a deficiency of centrobin intramanchette transport and/or intraflagellar transport (also microtubule-dependent) may result from a failure of centrobin-tubulin interaction determined by dimerization/multimerization of transgenic centrobin. In fact, it is known that over-expression of centrobin results in the formation of protein bundles ([10] and our unpublished data), a finding that may explain the tendency of over-expressed centrobin to accumulate in bulges along the sperm flagellum. By dissecting the conundrum of infertility rescue failure, biochemical, proteomic and transcriptomic analyses using the transgenic rats may be invaluable for providing additional insights into the mechanism of sperm decapitation and sperm flagellum abnormalities in the rat model as well as in equivalent phenotypes seen in the infertile human male.
